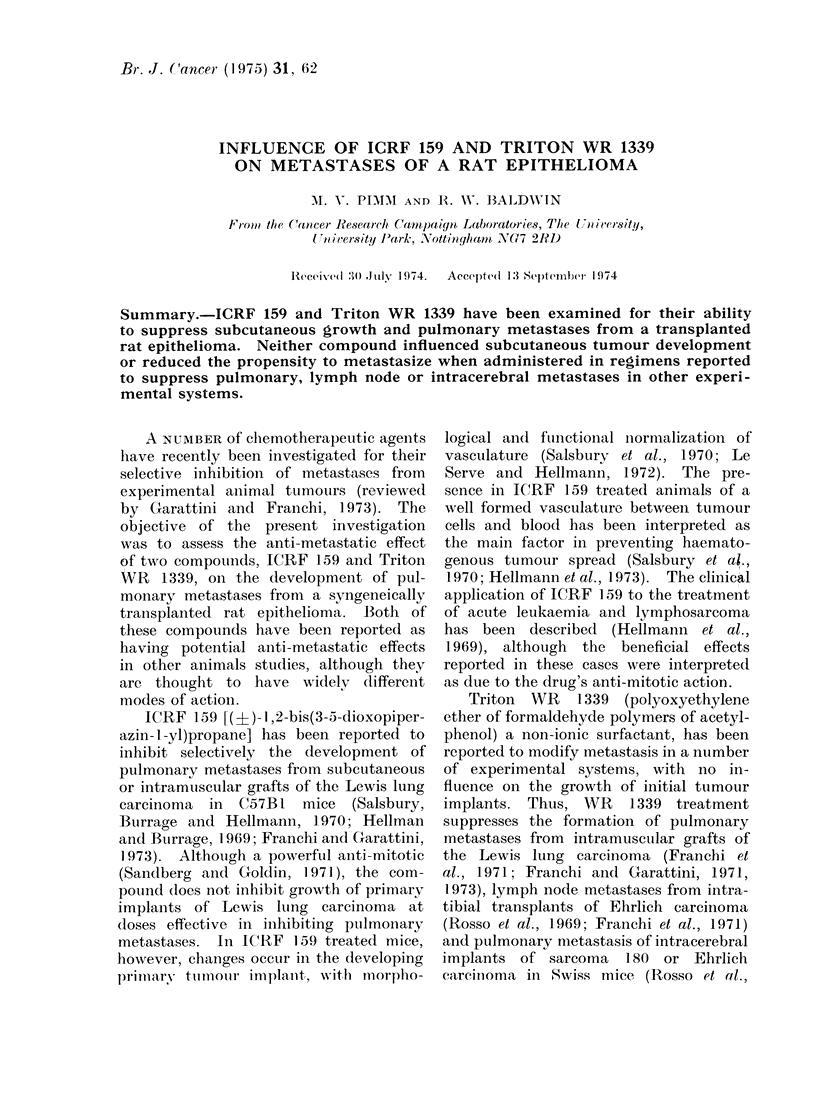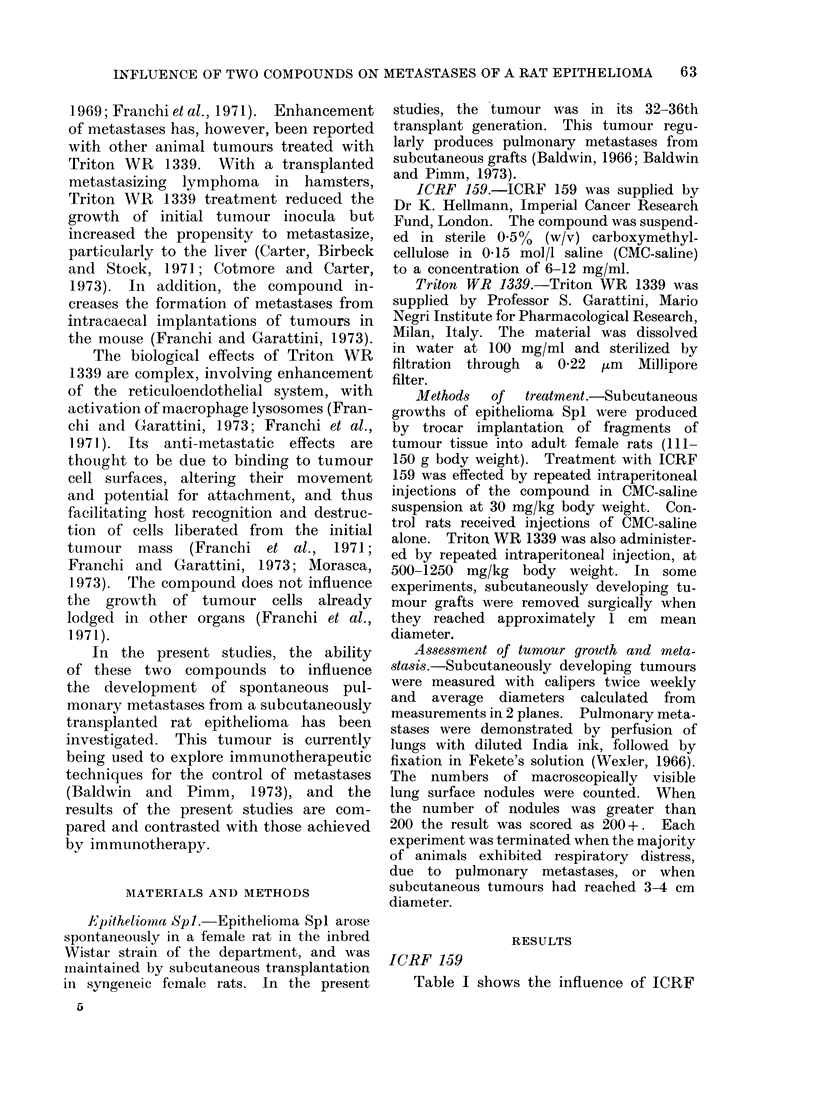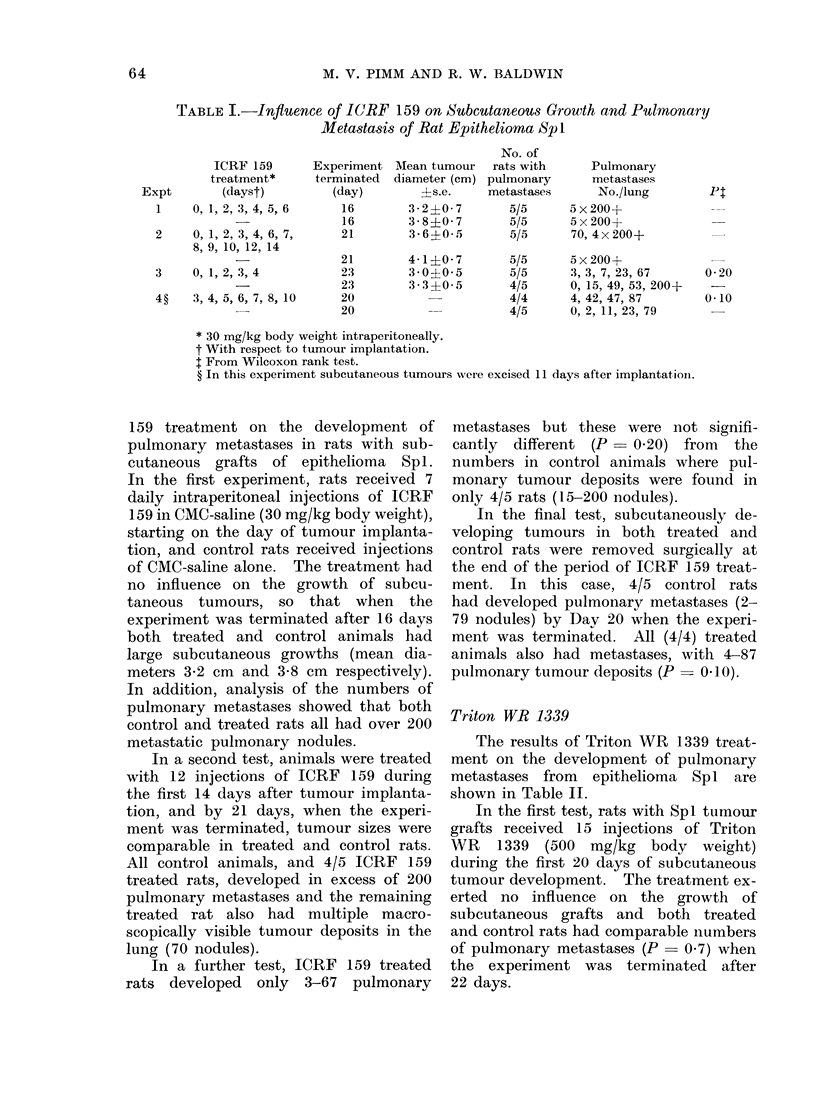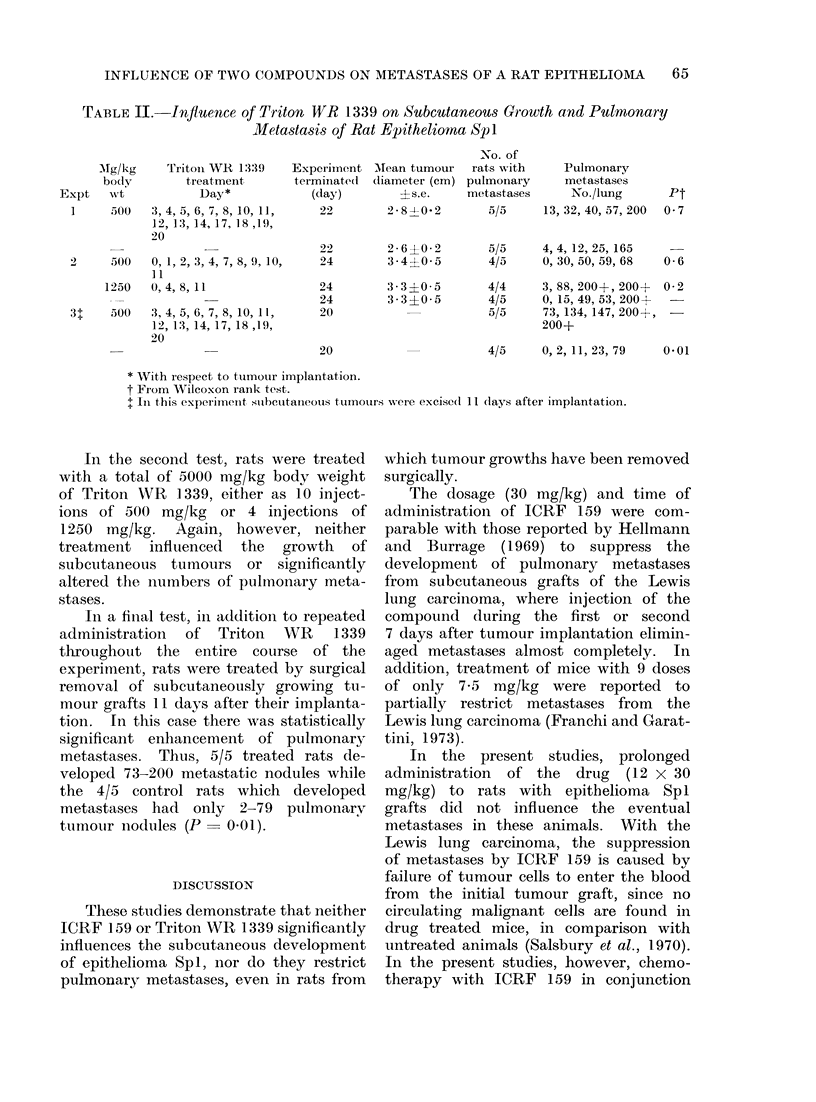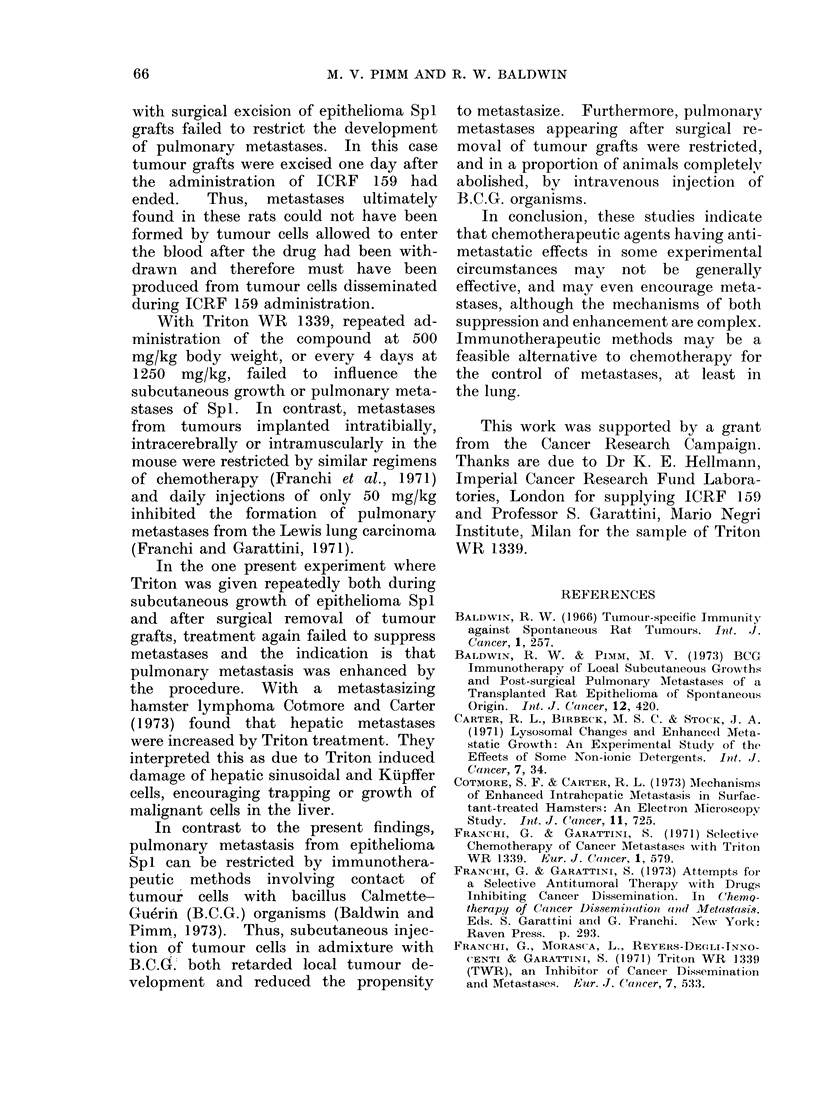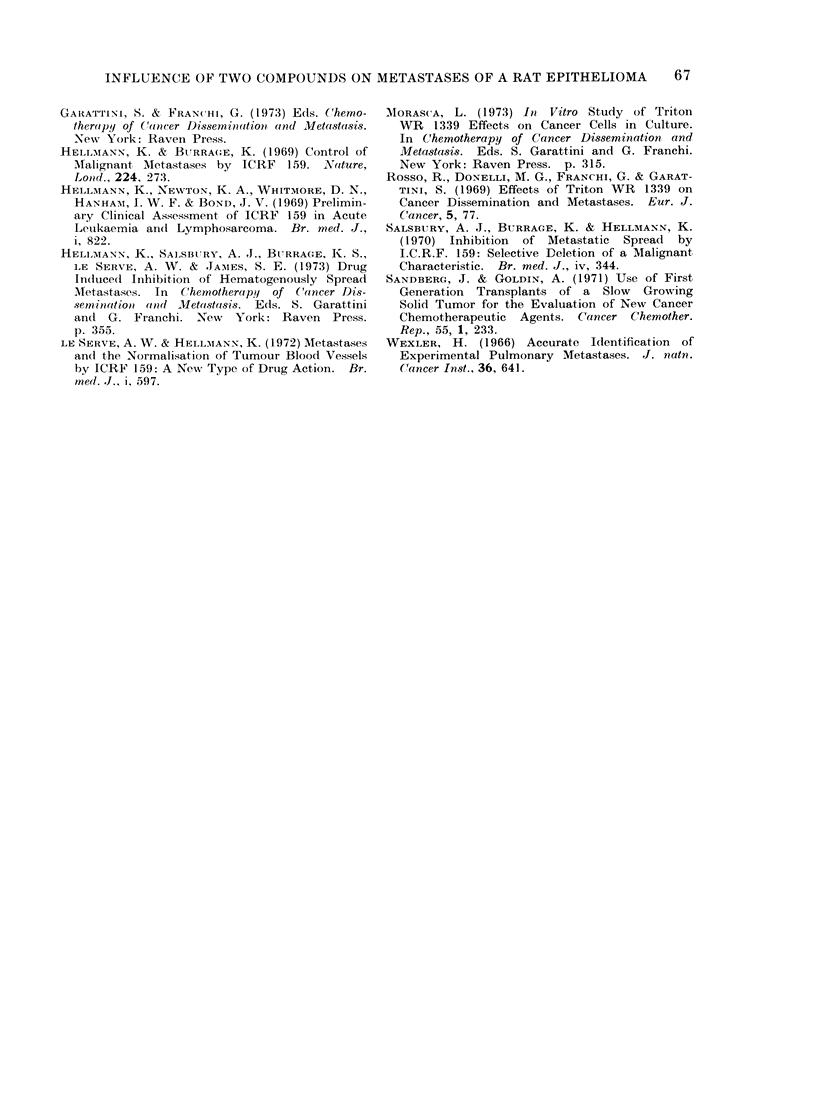# Influence of ICRF 159 and trition WR 1339 on metastases of a rat epithelioma.

**DOI:** 10.1038/bjc.1975.7

**Published:** 1975-01

**Authors:** M. V. Pimm, R. W. Baldwin

## Abstract

ICRF 159 and Triton WR 1339 have been examined for their ability to suppress subcutaneous growth and pulmonary metastases from a transplanted rat epithelioma. Neither compound influenced subcutaneous tumour development or reduced the propensity to metastasize when administered in regimens reported to suppress pulmonary, lymph node or intracerebral metastases in other experimental system.


*Br. J. Cancer* (1975) **31**, 62

### INFLUENCE OF ICRF 159 AND TRITON WR 1339 ON METASTASES OF A RAT EPITHELIOMA

M. V. PIMM AND R. W. BALDWIN

*From the Cancer Research Campaign Laboratories, The University, University Park, Nottingham NG7 2RD*

Received 30 July 1974. Accepted 13 September 1974

**Summary.—ICRF 159 and Triton WR 1339 have been examined for their ability to suppress subcutaneous growth and pulmonary metastases from a transplanted rat epithelioma. Neither compound influenced subcutaneous tumour development or reduced the propensity to metastasize when administered in regimens reported to suppress pulmonary, lymph node or intracerebral metastases in other experimental systems.**

A NUMBER of chemotherapeutic agents have recently been investigated for their selective inhibition of metastases from experimental animal tumours (reviewed by Garattini and Franchi, 1973). The objective of the present investigation was to assess the anti-metastatic effect of two compounds, ICRF 159 and Triton WR 1339, on the development of pulmonary metastases from a syngeneically transplanted rat epithelioma. Both of these compounds have been reported as having potential anti-metastatic effects in other animals studies, although they are thought to have widely different modes of action.

ICRF 159 [(±)-1,2-bis(3-5-dioxopiperazin-1-yl)propane] has been reported to inhibit selectively the development of pulmonary metastases from subcutaneous or intramuscular grafts of the Lewis lung carcinoma in C57Bl mice (Salsbury, Burrage and Hellmann, 1970; Hellman and Burrage, 1969; Franchi and Garattini, 1973). Although a powerful anti-mitotic (Sandberg and Goldin, 1971), the compound does not inhibit growth of primary implants of Lewis lung carcinoma at doses effective in inhibiting pulmonary metastases. In ICRF 159 treated mice, however, changes occur in the developing primary tumour implant, with morphological and functional normalization of vasculature (Salsbury *et al.*, 1970; Le Serve and Hellmann, 1972). The presence in ICRF 159 treated animals of a well formed vasculature between tumour cells and blood has been interpreted as the main factor in preventing haematogenous tumour spread (Salsbury *et al.*, 1970; Hellmann *et al.*, 1973). The clinical application of ICRF 159 to the treatment of acute leukaemia and lymphosarcoma has been described (Hellmann *et al.*, 1969), although the beneficial effects reported in these cases were interpreted as due to the drug's anti-mitotic action.

Triton WR 1339 (polyoxyethylene ether of formaldehyde polymers of acetylphenol) a non-ionic surfactant, has been reported to modify metastasis in a number of experimental systems, with no influence on the growth of initial tumour implants. Thus, WR 1339 treatment suppresses the formation of pulmonary metastases from intramuscular grafts of the Lewis lung carcinoma (Franchi *et al.*, 1971; Franchi and Garattini, 1971, 1973), lymph node metastases from intratibial transplants of Ehrlich carcinoma (Rosso *et al.*, 1969; Franchi *et al.*, 1971) and pulmonary metastasis of intracerebral implants of sarcoma 180 or Ehrlich carcinoma in Swiss mice (Rosso *et al.*,

1969; Franchi *et al.*, 1971). Enhancement of metastases has, however, been reported with other animal tumours treated with Triton WR 1339. With a transplanted metastasizing lymphoma in hamsters, Triton WR 1339 treatment reduced the growth of initial tumour inocula but increased the propensity to metastasize, particularly to the liver (Carter, Birbeck and Stock, 1971; Cotmore and Carter, 1973). In addition, the compound increases the formation of metastases from intracaecal implantations of tumours in the mouse (Franchi and Garattini, 1973).

The biological effects of Triton WR 1339 are complex, involving enhancement of the reticuloendothelial system, with activation of macrophage lysosomes (Franchi and Garattini, 1973; Franchi *et al.*, 1971). Its anti-metastatic effects are thought to be due to binding to tumour cell surfaces, altering their movement and potential for attachment, and thus facilitating host recognition and destruction of cells liberated from the initial tumour mass (Franchi *et al.*, 1971; Franchi and Garattini, 1973; Morasca, 1973). The compound does not influence the growth of tumour cells already lodged in other organs (Franchi *et al.*, 1971).

In the present studies, the ability of these two compounds to influence the development of spontaneous pulmonary metastases from a subcutaneously transplanted rat epithelioma has been investigated. This tumour is currently being used to explore immunotherapeutic techniques for the control of metastases (Baldwin and Pimm, 1973), and the results of the present studies are compared and contrasted with those achieved by immunotherapy.

### MATERIALS AND METHODS

*Epithelioma Sp1.*—Epithelioma Sp1 arose spontaneously in a female rat in the inbred Wistar strain of the department, and was maintained by subcutaneous transplantation in syngeneic female rats. In the present studies, the tumour was in its 32–36th transplant generation. This tumour regularly produces pulmonary metastases from subcutaneous grafts (Baldwin, 1966; Baldwin and Pimm, 1973).

*ICRF 159.*—ICRF 159 was supplied by Dr K. Hellmann, Imperial Cancer Research Fund, London. The compound was suspended in sterile 0·5% (w/v) carboxymethylcellulose in 0·15 mol/l saline (CMC-saline) to a concentration of 6–12 mg/ml.

*Triton WR 1339.*—Triton WR 1339 was supplied by Professor S. Garattini, Mario Negri Institute for Pharmacological Research, Milan, Italy. The material was dissolved in water at 100 mg/ml and sterilized by filtration through a 0·22 $\mu$m Millipore filter.

*Methods of treatment.*—Subcutaneous growths of epithelioma Sp1 were produced by trocar implantation of fragments of tumour tissue into adult female rats (111–150 g body weight). Treatment with ICRF 159 was effected by repeated intraperitoneal injections of the compound in CMC-saline suspension at 30 mg/kg body weight. Control rats received injections of CMC-saline alone. Triton WR 1339 was also administered by repeated intraperitoneal injection, at 500–1250 mg/kg body weight. In some experiments, subcutaneously developing tumour grafts were removed surgically when they reached approximately 1 cm mean diameter.

*Assessment of tumour growth and metastasis.*—Subcutaneously developing tumours were measured with calipers twice weekly and average diameters calculated from measurements in 2 planes. Pulmonary metastases were demonstrated by perfusion of lungs with diluted India ink, followed by fixation in Fekete's solution (Wexler, 1966). The numbers of macroscopically visible lung surface nodules were counted. When the number of nodules was greater than 200 the result was scored as 200+. Each experiment was terminated when the majority of animals exhibited respiratory distress, due to pulmonary metastases, or when subcutaneous tumours had reached 3–4 cm diameter.

### RESULTS

### *ICRF 159*

Table I shows the influence of ICRF

TABLE I.—*Influence of ICRF* 159 *on Subcutaneous Growth and Pulmonary Metastasis of Rat Epithelioma Sp*1

| Expt | ICRF 159 treatment* (days†) | Experiment terminated (day) | Mean tumour diameter (cm) ±s.e. | No. of rats with pulmonary metastases | Pulmonary metastases No./lung | P‡ |
|---|---|---|---|---|---|---|
| 1 | 0, 1, 2, 3, 4, 5, 6 | 16 | 3·2±0·7 | 5/5 | 5×200+ | — |
| | — | 16 | 3·8±0·7 | 5/5 | 5×200+ | — |
| 2 | 0, 1, 2, 3, 4, 6, 7, 8, 9, 10, 12, 14 | 21 | 3·6±0·5 | 5/5 | 70, 4×200+ | — |
| | — | 21 | 4·1±0·7 | 5/5 | 5×200+ | — |
| 3 | 0, 1, 2, 3, 4 | 23 | 3·0±0·5 | 5/5 | 3, 3, 7, 23, 67 | 0·20 |
| | — | 23 | 3·3±0·5 | 4/5 | 0, 15, 49, 53, 200+ | — |
| 4§ | 3, 4, 5, 6, 7, 8, 10 | 20 | — | 4/4 | 4, 42, 47, 87 | 0·10 |
| | — | 20 | — | 4/5 | 0, 2, 11, 23, 79 | — |

* 30 mg/kg body weight intraperitoneally.
† With respect to tumour implantation.
‡ From Wilcoxon rank test.
§ In this experiment subcutaneous tumours were excised 11 days after implantation.

159 treatment on the development of pulmonary metastases in rats with subcutaneous grafts of epithelioma Sp1. In the first experiment, rats received 7 daily intraperitoneal injections of ICRF 159 in CMC-saline (30 mg/kg body weight), starting on the day of tumour implantation, and control rats received injections of CMC-saline alone. The treatment had no influence on the growth of subcutaneous tumours, so that when the experiment was terminated after 16 days both treated and control animals had large subcutaneous growths (mean diameters 3·2 cm and 3·8 cm respectively). In addition, analysis of the numbers of pulmonary metastases showed that both control and treated rats all had over 200 metastatic pulmonary nodules.

In a second test, animals were treated with 12 injections of ICRF 159 during the first 14 days after tumour implantation, and by 21 days, when the experiment was terminated, tumour sizes were comparable in treated and control rats. All control animals, and 4/5 ICRF 159 treated rats, developed in excess of 200 pulmonary metastases and the remaining treated rat also had multiple macroscopically visible tumour deposits in the lung (70 nodules).

In a further test, ICRF 159 treated rats developed only 3–67 pulmonary metastases but these were not significantly different ($P = 0{\cdot}20$) from the numbers in control animals where pulmonary tumour deposits were found in only 4/5 rats (15–200 nodules).

In the final test, subcutaneously developing tumours in both treated and control rats were removed surgically at the end of the period of ICRF 159 treatment. In this case, 4/5 control rats had developed pulmonary metastases (2–79 nodules) by Day 20 when the experiment was terminated. All (4/4) treated animals also had metastases, with 4–87 pulmonary tumour deposits ($P = 0{\cdot}10$).

### *Triton WR 1339*

The results of Triton WR 1339 treatment on the development of pulmonary metastases from epithelioma Sp1 are shown in Table II.

In the first test, rats with Sp1 tumour grafts received 15 injections of Triton WR 1339 (500 mg/kg body weight) during the first 20 days of subcutaneous tumour development. The treatment exerted no influence on the growth of subcutaneous grafts and both treated and control rats had comparable numbers of pulmonary metastases ($P = 0{\cdot}7$) when the experiment was terminated after 22 days.

TABLE II.—*Influence of Triton WR* 1339 *on Subcutaneous Growth and Pulmonary Metastasis of Rat Epithelioma Sp*1

| Expt | Mg/kg body wt | Triton WR 1339 treatment Day* | Experiment terminated (day) | Mean tumour diameter (cm) ±s.e. | No. of rats with pulmonary metastases | Pulmonary metastases No./lung | P† |
|---|---|---|---|---|---|---|---|
| 1 | 500 | 3, 4, 5, 6, 7, 8, 10, 11, 12, 13, 14, 17, 18 ,19, 20 | 22 | 2·8±0·2 | 5/5 | 13, 32, 40, 57, 200 | 0·7 |
| — | — | — | 22 | 2·6±0·2 | 5/5 | 4, 4, 12, 25, 165 | — |
| 2 | 500 | 0, 1, 2, 3, 4, 7, 8, 9, 10, 11 | 24 | 3·4±0·5 | 4/5 | 0, 30, 50, 59, 68 | 0·6 |
| | 1250 | 0, 4, 8, 11 | 24 | 3·3±0·5 | 4/4 | 3, 88, 200+, 200+ | 0·2 |
| | — | — | 24 | 3·3±0·5 | 4/5 | 0, 15, 49, 53, 200+ | — |
| 3‡ | 500 | 3, 4, 5, 6, 7, 8, 10, 11, 12, 13, 14, 17, 18 ,19, 20 | 20 | — | 5/5 | 73, 134, 147, 200+, 200+ | — |
| | — | — | 20 | — | 4/5 | 0, 2, 11, 23, 79 | 0·01 |

* With respect to tumour implantation.
† From Wilcoxon rank test.
‡ In this experiment subcutaneous tumours were excised 11 days after implantation.

In the second test, rats were treated with a total of 5000 mg/kg body weight of Triton WR 1339, either as 10 injections of 500 mg/kg or 4 injections of 1250 mg/kg. Again, however, neither treatment influenced the growth of subcutaneous tumours or significantly altered the numbers of pulmonary metastases.

In a final test, in addition to repeated administration of Triton WR 1339 throughout the entire course of the experiment, rats were treated by surgical removal of subcutaneously growing tumour grafts 11 days after their implantation. In this case there was statistically significant enhancement of pulmonary metastases. Thus, 5/5 treated rats developed 73–200 metastatic nodules while the 4/5 control rats which developed metastases had only 2–79 pulmonary tumour nodules ($P = 0{\cdot}01$).

### DISCUSSION

These studies demonstrate that neither ICRF 159 or Triton WR 1339 significantly influences the subcutaneous development of epithelioma Sp1, nor do they restrict pulmonary metastases, even in rats from which tumour growths have been removed surgically.

The dosage (30 mg/kg) and time of administration of ICRF 159 were comparable with those reported by Hellmann and Burrage (1969) to suppress the development of pulmonary metastases from subcutaneous grafts of the Lewis lung carcinoma, where injection of the compound during the first or second 7 days after tumour implantation eliminaged metastases almost completely. In addition, treatment of mice with 9 doses of only 7·5 mg/kg were reported to partially restrict metastases from the Lewis lung carcinoma (Franchi and Garattini, 1973).

In the present studies, prolonged administration of the drug ($12 \times 30$ mg/kg) to rats with epithelioma Sp1 grafts did not influence the eventual metastases in these animals. With the Lewis lung carcinoma, the suppression of metastases by ICRF 159 is caused by failure of tumour cells to enter the blood from the initial tumour graft, since no circulating malignant cells are found in drug treated mice, in comparison with untreated animals (Salsbury *et al.*, 1970). In the present studies, however, chemotherapy with ICRF 159 in conjunction with surgical excision of epithelioma Sp1 grafts failed to restrict the development of pulmonary metastases. In this case tumour grafts were excised one day after the administration of ICRF 159 had ended. Thus, metastases ultimately found in these rats could not have been formed by tumour cells allowed to enter the blood after the drug had been withdrawn and therefore must have been produced from tumour cells disseminated during ICRF 159 administration.

With Triton WR 1339, repeated administration of the compound at 500 mg/kg body weight, or every 4 days at 1250 mg/kg, failed to influence the subcutaneous growth or pulmonary metastases of Sp1. In contrast, metastases from tumours implanted intratibially, intracerebrally or intramuscularly in the mouse were restricted by similar regimens of chemotherapy (Franchi *et al.*, 1971) and daily injections of only 50 mg/kg inhibited the formation of pulmonary metastases from the Lewis lung carcinoma (Franchi and Garattini, 1971).

In the one present experiment where Triton was given repeatedly both during subcutaneous growth of epithelioma Sp1 and after surgical removal of tumour grafts, treatment again failed to suppress metastases and the indication is that pulmonary metastasis was enhanced by the procedure. With a metastasizing hamster lymphoma Cotmore and Carter (1973) found that hepatic metastases were increased by Triton treatment. They interpreted this as due to Triton induced damage of hepatic sinusoidal and Küpffer cells, encouraging trapping or growth of malignant cells in the liver.

In contrast to the present findings, pulmonary metastasis from epithelioma Sp1 can be restricted by immunotherapeutic methods involving contact of tumour cells with bacillus Calmette–Guérin (B.C.G.) organisms (Baldwin and Pimm, 1973). Thus, subcutaneous injection of tumour cells in admixture with B.C.G. both retarded local tumour development and reduced the propensity to metastasize. Furthermore, pulmonary metastases appearing after surgical removal of tumour grafts were restricted, and in a proportion of animals completely abolished, by intravenous injection of B.C.G. organisms.

In conclusion, these studies indicate that chemotherapeutic agents having antimetastatic effects in some experimental circumstances may not be generally effective, and may even encourage metastases, although the mechanisms of both suppression and enhancement are complex. Immunotherapeutic methods may be a feasible alternative to chemotherapy for the control of metastases, at least in the lung.

This work was supported by a grant from the Cancer Research Campaign. Thanks are due to Dr K. E. Hellmann, Imperial Cancer Research Fund Laboratories, London for supplying ICRF 159 and Professor S. Garattini, Mario Negri Institute, Milan for the sample of Triton WR 1339.

### REFERENCES

BALDWIN, R. W. (1966) Tumour-specific Immunity against Spontaneous Rat Tumours. *Int. J. Cancer*, **1**, 257.

BALDWIN, R. W. & PIMM, M. V. (1973) BCG Immunotherapy of Local Subcutaneous Growths and Post-surgical Pulmonary Metastases of a Transplanted Rat Epithelioma of Spontaneous Origin. *Int. J. Cancer*, **12**, 420.

CARTER, R. L., BIRBECK, M. S. C. & STOCK, J. A. (1971) Lysosomal Changes and Enhanced Metastatic Growth: An Experimental Study of the Effects of Some Non-ionic Detergents. *Int. J. Cancer*, **7**, 34.

COTMORE, S. F. & CARTER, R. L. (1973) Mechanisms of Enhanced Intrahepatic Metastasis in Surfactant-treated Hamsters: An Electron Microscopy Study. *Int. J. Cancer*, **11**, 725.

FRANCHI, G. & GARATTINI, S. (1971) Selective Chemotherapy of Cancer Metastases with Triton WR 1339. *Eur. J. Cancer*, **1**, 579.

FRANCHI, G. & GARATTINI, S. (1973) Attempts for a Selective Antitumoral Therapy with Drugs Inhibiting Cancer Dissemination. In *Chemotherapy of Cancer Dissemination and Metastasis*. Eds. S. Garattini and G. Franchi. New York: Raven Press. p. 293.

FRANCHI, G., MORASCA, L., REYERS-DEGLI-INNOCENTI & GARATTINI, S. (1971) Triton WR 1339 (TWR), an Inhibitor of Cancer Dissemination and Metastases. *Eur. J. Cancer*, **7**, 533.

GARATTINI, S. & FRANCHI, G. (1973) Eds. *Chemotherapy of Cancer Dissemination and Metastasis.* New York: Raven Press.

HELLMANN, K. & BURRAGE, K. (1969) Control of Malignant Metastases by ICRF 159. *Nature, Lond.*, **224**, 273.

HELLMANN, K., NEWTON, K. A., WHITMORE, D. N., HANHAM, I. W. F. & BOND, J. V. (1969) Preliminary Clinical Assessment of ICRF 159 in Acute Leukaemia and Lymphosarcoma. *Br. med. J.*, i, 822.

HELLMANN, K., SALSBURY, A. J., BURRAGE, K. S., LE SERVE, A. W. & JAMES, S. E. (1973) Drug Induced Inhibition of Hematogenously Spread Metastases. In *Chemotherapy of Cancer Dissemination and Metastasis.* Eds. S. Garattini and G. Franchi. New York: Raven Press. p. 355.

LE SERVE, A. W. & HELLMANN, K. (1972) Metastases and the Normalisation of Tumour Blood Vessels by ICRF 159: A New Type of Drug Action. *Br. med. J.*, i, 597.

MORASCA, L. (1973) *In Vitro* Study of Triton WR 1339 Effects on Cancer Cells in Culture. In *Chemotherapy of Cancer Dissemination and Metastasis.* Eds. S. Garattini and G. Franchi. New York: Raven Press. p. 315.

ROSSO, R., DONELLI, M. G., FRANCHI, G. & GARATTINI, S. (1969) Effects of Triton WR 1339 on Cancer Dissemination and Metastases. *Eur. J. Cancer*, **5**, 77.

SALSBURY, A. J., BURRAGE, K. & HELLMANN, K. (1970) Inhibition of Metastatic Spread by I.C.R.F. 159: Selective Deletion of a Malignant Characteristic. *Br. med. J.*, iv, 344.

SANDBERG, J. & GOLDIN, A. (1971) Use of First Generation Transplants of a Slow Growing Solid Tumor for the Evaluation of New Cancer Chemotherapeutic Agents. *Cancer Chemother. Rep.*, 55, **1**, 233.

WEXLER, H. (1966) Accurate Identification of Experimental Pulmonary Metastases. *J. natn. Cancer Inst.*, **36**, 641.